# *Wolbachia*-Based Dengue Virus Inhibition Is Not Tissue-Specific in *Aedes aegypti*

**DOI:** 10.1371/journal.pntd.0005145

**Published:** 2016-11-17

**Authors:** Hilaria E. Amuzu, Elizabeth A. McGraw

**Affiliations:** School of Biological Sciences, Monash University, Clayton, Victoria, Australia; Colorado State University, UNITED STATES

## Abstract

**Background:**

Dengue fever, caused by the dengue virus (DENV), is now the most common arbovirus transmitted disease globally. One novel approach to control DENV is to use the endosymbiotic bacterium, *Wolbachia pipientis*, to limit DENV replication inside the primary mosquito vector, *Aedes aegypti*. *Wolbachia* that is naturally present in a range of insects reduces the capacity for viruses, bacteria, parasites and fungi to replicate inside insects. *Wolbachia’s* mode of action is not well understood but may involve components of immune activation or competition with pathogens for limited host resources. The strength of *Wolbachia*-based anti DENV effects appear to correlate with bacterial density in the whole insect and in cell culture. Here we aimed to determine whether particular tissues, especially those with high *Wolbachia* densities or immune activity, play a greater role in mediating the anti DENV effect.

**Methodology/findings:**

*Ae*. *aegypti* mosquito lines with and without *Wolbachia* (Wildtype) were orally fed DENV 3 and their viral loads subsequently measured over two time points post infection in the midgut, head, salivary glands, Malpighian tubules, fat body and carcass. We did not find correlations between *Wolbachia* densities and DENV loads in any tissue, nor with DENV loads in salivary glands, the endpoint of infection. This is in contrast with strong positive correlations between DENV loads in a range of tissues and salivary gland loads for Wildtype mosquitoes. Lastly, there was no evidence of a heightened role for tissues with known immune function including the fat body and the Malpighian tubules in *Wolbachia*’s limitation of DENV.

**Conclusion/significance:**

We conclude that the efficacy of DENV blocking in *Wolbachia* infected mosquitoes is not reliant on any particular tissue. This work therefore suggests that the mechanism of *Wolbachia*-based antiviral effects is either systemic or acts locally via processes that are fundamental to diverse cell types. We further conclude that the relationship between DENV blocking and *Wolbachia* density is not linear in mosquito tissues

## Author Summary

Dengue fever caused by the dengue virus (DENV) is transmitted by the mosquito, *Aedes aegypti*. To control the disease, an intracellular bacterium called *Wolbachia* has been introduced into *Ae*. *aegypti* where it blocks/limits success of infection of DENV. The mechanistic basis of blocking is not well understood but may involve *Wolbachia* activating the host immune system or competing with DENV for host resources. The strength of blocking appears to correlate with *Wolbachia* density. Here, we aimed to determine if any particular tissues inside the mosquito play a greater role in blocking. Tissues were chosen based on their *Wolbachia* density and their roles in infection and immunity. *Wolbachia* infected and uninfected mosquitoes were orally infected with DENV and *Wolbachia* density and DENV load were assessed in midgut, salivary gland, head, Malpighian tubules, fat body and carcass. *Wolbachia* density did not correlate with DENV loads in the same tissues nor with DENV loads in the salivary glands. We also showed that no one tissue appeared to play a greater role in blocking. In summary, these finding suggest that in the mosquito a threshold *Wolbachia* density may be required for DENV blocking. Our findings also suggest that blocking may involve mechanisms that are fundamental to all cells.

## Introduction

Dengue fever, caused by the dengue virus (DENV), is the most prevalent arthropod transmitted virus, endemic in over 100 countries [1,2].The virus is comprised of four antigenically distinct serotypes (1–4) [3,4]. DENV is transmitted by *Aedes aegypti* and *Ae*. *albopictus* with the former being the principal vector [5]. With no specific antiviral drugs, management of the disease has mainly relied on relieving the associated symptoms of fever, headache and rash [6]. As the current tetravalent dengue vaccine offers incomplete protection [7], vector control remains the primary means of reducing disease prevalence.

One example of an emerging vector control strategy involves the use of a bacterial endosymbiont, *Wolbachia pipenti*s that is naturally present in 40% of arthropods [8] and 28% of mosquito species, including *Ae*. *albopictus*, and *Ae*. *notoscriptus*. Interestingly, *Ae*. *aegypti* is not naturally infected with the symbiont [9]. Over the last decade, three different *Wolbachia* strains have been transinfected into *Ae*. *aegypti* where they form stable, inherited infections including; *w*MelPop-CLA and *w*Mel, both from *Drosophila melanogaster*, *w*AlbB from *Ae*. *Albopictus* and *w*Mel*w*AlbB, which is a superinfection from both host donors [10–13]. In these mosquito vectors, *Wolbachia* demonstrates an ability to limit or “block” the success of infection by viruses, nematodes and parasites [14–16]. This effect forms the basis of *Wolbachia*-based biocontrol trials to interrupt disease transmission in the human population via the vector [17]. The most advanced of such trials are focused on DENV control where the *w*Mel strain has been released into wild *Ae*. *aegypti* mosquitoes and successfully spread [18].

Despite widespread field-testing, the mechanistic basis of *Wolbachia*-DENV blocking is poorly understood. Pathogen blocking has been partly attributed to the ability of the bacterium to increase the basal immune activity of the host thereby enabling it to resist subsequent DENV infection in a process known as ‘immune priming’ [19–21]. *Wolbachia*-DENV inhibition may also be as a result of competition between the symbiont and viruses for vital host nutrients such as cholesterol, as demonstrated in *Drosophila* [22]. Such competition may be expected given that the *Wolbachia* genome lacks a range of key genes in lipid biosynthesis pathways [23] and because viruses are heavily reliant on host cholesterol for replication [24,25]. Neither immune priming nor cholesterol competition however, can completely explain *Wolbachia*-DENV blocking.

The strength of blocking appears to correlate with *Wolbachia* density, whereby higher densities of the symbiont are associated with greater viral inhibition [11,26–28]. In mosquito cell lines, only highly infected cells show almost complete DENV inhibition [27,28]. The same relationship has been documented in other insects. In *Drosophila simulans*, the *w*Mel, *w*Au and *w*Ri strains grow to high densities and provide protection against *Drosophila* C virus (DCV). In contrast, the *w*Ha and *w*No strains that grow to very low densities show little blocking [29]. The fact that *w*AlbB is unable to block DENV in its natural host *Ae*. *albopictus* has also been attributed to low symbiont numbers. In *Ae*. *aegypti* where *w*AlbB has been introduced and hence grows to higher densities, DENV blocking is much stronger [28]. The correlation is further shown in *Ae*. *aegypti* by the disparity in blocking between the virulent *w*MelPop-CLA strain, which grows to very high densities compared to the *w*Mel strain which grows to moderate densities [11].

Several studies have reported that *Wolbachia* is found at different densities in various tissues of the mosquito body, with the ovaries and Malpighian tubules tending to have high densities [14,21,28,30,31]. Osborne *et al*., [32] have suggested that *Wolbachia* density within the head, gut and Malpighian tubules correlated with the ability to mediate protection against DCV in *D*. *simulans*. These different tissues may be of varying importance for pathogen blocking as predicted by their *Wolbachia* densities or if they play a particular functional role in *Wolbachia*-based pathogen blocking. For example, the fat body is mainly involved in pathogen defence [33,34] and the Malpighian tubules, that happen to have very high *Wolbachia* densities now appear to have immune function [35]. It is unknown if there is a correlation between the *Wolbachia* encountered by DENV in these tissues and the subsequent progression of infection to the salivary glands as the endpoint of transmission.

When a mosquito takes a viremic blood meal, the virus first infects the midgut and then it disseminates to other tissues such as the Malpighian tubules, fat body, trachea and the salivary glands, where it can be transmitted to a human via the saliva on a subsequent bite [5]. The rate of DENV transmission correlates with the titre of virus in the salivary glands when studied in animal models [36] and mosquito infection rate is also known to correlate with virus infectious dose [37]. Even though several studies have suggested that intermediate mosquito tissues are infected by the virus differentially over time [38–41], it is not clear if there is a correlation between DENV infection in these tissues and that in the salivary glands.

Here we have examined the infectivity and viral load of a DENV serotype 3 strain in the tissues of Wildtype and *w*Mel-infected *Ae*. *aegypti*. Specifically we have assessed whether *Wolbachia* densities predict DENV load in the same tissue and if densities in intermediate tissues predict subsequent DENV loads in the salivary glands. We found that there was a positive correlation between DENV loads in intermediate tissues and salivary glands in Wildtype but not *Wolbachia*-infected mosquitoes. There was also no correlation between *Wolbachia* densities and DENV loads in any particular tissue. Together, these findings suggest that no one tissue is particularly important for *Wolbachia*-based blocking and that *Wolbachia* may simply be limiting virus at the level of each individual cell, by fundamental processes shared by diverse cell types.

## Materials and Methods

### Mosquito rearing

Two mosquito lines were used for this experiment; *Wolbachia* infected [11] and *Wolbachia* uninfected *Ae*. *aegypti* mosquitoes designated *w*Mel.F and Wildtype [31,42], respectively. The *w*Mel.F mosquito line was collected in 2012 from field release sites in Cairns, Australia [18] while the Wildtype line was collected in 2014 from Babinda, Australia. The Wildtype mosquito line was used within four generations of field collection to limit inbreeding. At every generation, the *w*Mel.F mosquito line was outcrossed with 20% Wildtype males to prevent genetic drift between the two lines. Adult mosquitoes were maintained on 10% sucrose while the larvae were fed TetraMin® fish food (Melle, Germany) *ad libitum*. Mosquitoes were reared under standard conditions of 25°C temperature, 65% relative humidity and photoperiod 12 hours light: dark.

### Oral infection of mosquitoes with DENV 3

The DENV 3 strain used for this experiment was sampled from a patient during an outbreak in Cairns, Australia in 2008/2009 [43]. This strain was selected because it caused one of the largest dengue outbreaks in Australia [43] and because it has been demonstrated to infect both *w*Mel and Wildtype mosquitoes at a high rate [44]. Passage 6 of DENV 3 (PFU 10^6^) was propagated using the protocol by Ye *et al*., [45] and stored in single use aliquots of 1mL at -80°C. The virus was mixed with defibrinated sheep’s blood in the ratio 1:1 and fed through a membrane feeder to three to five day old mosquitoes. The mosquitoes were starved for 24 hours prior to oral infection. The *w*Mel.F and Wildtype mosquitoes were both fed simultaneously over a period of three hours [44]. Mosquitoes were then anesthetized on ice and females that did not feed were sorted out and discarded. Engorged mosquitoes were maintained on 10% sucrose at 25°C until they were dissected.

### Dissection of tissues

The midguts, salivary glands, head, fat body, Malpighian tubules and carcass were dissected from each individual mosquito. These tissues were chosen mainly based on their functional role in DENV infection, dissemination and transmission in the mosquito. DENV first infects and replicates in the midgut before being disseminated to other tissues [5]. The end point of disseminated DENV is the salivary glands from where it is transmitted to the human host through the saliva when the mosquito takes a blood meal [5]. Assessment of DENV dissemination in mosquitoes is commonly done using the head tissue given ease of dissection [21,42]. Tissues were dissected on 8 and 14 days post infection (dpi). These time points were chosen to reflect the early stage of infection where DENV would have disseminated from the midgut to other tissues and the late stage of infection where infection would have been well established [44]. Dissections were done in 1X phosphate buffered saline (PBS). Tissues of each individual mosquito were placed in 96-well PCR plates (VWR LabAdvantage, Australia) containing 200*ul* of extraction buffer (0.01M Trizma base, 0.001M EDTA, 0.05M NaCl and 2.5*ul* proteinase K) and 2-mm-diameter glass beads (Merck KGaA, Darmstadt, Germany). Ovaries were separated from the carcass and discarded to ensure that *Wolbachia* density was not unduly influenced by gravid females. To minimize contamination within mosquito lines the dissecting pins were immersed in 80% ethanol for ~10 seconds between individual mosquitoes and discarded after every 20 individuals. New dissecting pins were used for each line to avoid cross contamination between Wildtype and *w*Mel.F mosquitoes. All tissues were stored at -80°C prior to RNA/DNA co-extraction. The entire experiment was replicated three times.

### RNA/DNA extraction

Plates containing dissected tissues were homogenized for 1 min 30 seconds in a mini-Beadbeater (BioSpec Products, Bartlesville, OK). They were then incubated in a thermo cycler (C1000^Tm^ Thermal cycler, Bio-Rad, California USA) at 56°C for 5 min, then 98°C for 5 min for the simultaneous extraction of RNA and DNA. The extracted RNA/DNA was stored at -80°C and subsequently used for the quantification of DENV 3 RNA copies and *Wolbachia* density.

### Quantification of DENV 3 RNA copies

Taqman qPCR was used to quantify DENV 3 RNA copies in LightCycler480 (Roche, Applied Science, Switzerland). The RealTime Ready RNA Virus Master (^©^1996–2016 Roche Diagnostics) was used for concurrent cDNA synthesis and DENV 3 RNA copies quantification following manufacturer’s protocol. Primers for DENV were designed from the 3’UTR region with HEX labelled probes [46]. The following qPCR cycling conditions were used: reverse transcription at 50°C for 10 min, initial denaturation at 95°C for 30s, 45cycles of amplification at 95°C for 5s and 60°C for 30s and a final cooling step at 40°C for 10s. Absolute quantification of DENV 3 RNA copies for individual tissues was extrapolated from a standard curve as previously reported [14].

### Quantification of *Wolbachia* density

Taqman multiplex qPCR was used for the quantification of the WD0513 *Wolbachia* gene [47] in LightCycler480 (Roche, Applied Science, Switzerland). The WD0513 gene was normalised to the mosquito housekeeping gene RPS17 [48,49] to account for different tissue sizes. The qPCR cycling conditions used are as follows: An initial incubation at 90°C for 5min followed by 45 cycles of amplification at 95°C for 10s, 60°C for 15s and 72°C for 1s and a final cooling step of 40°C for 10s. Relative quantification of *Wolbachia* was done using the inbuilt algorithm of LightCycler480.

### Data analysis

Tissue infectivity (proportion infected) of DENV 3 was analysed using the binary logistic function in a generalized linear model with presence or absence of DENV 3 infection as the response variable and tissue type and time as predicting factors. DENV 3 RNA copies (DENV load) in tissues was analysed using the tweedie distribution with log link function in a generalized linear model with DENV load as the response variable and tissue type and time as predicting factors. *Wolbachia* density in tissues was analysed using the tweedie distribution with log link function in a generalized linear model with *Wolbachia* density as the response variable and time and tissue as the predictive factors. Models were run separately for the Wildtype and *w*Mel.F mosquito lines. Non-Parametric Spearman correlation co-efficient was used to test for correlation between the following: (1) DENV loads in intermediate tissues and salivary glands, (2) *Wolbachia* density in tissues and DENV load in salivary glands and (3) DENV load and *Wolbachia* density in the same tissue. All statistical analyses were performed in SPSS^®^ (IBM SPSS Statistics for Windows, Version 20.0. Armonk, NY)

## Results

### DENV infectivity by tissue over time

**Fig 1:**
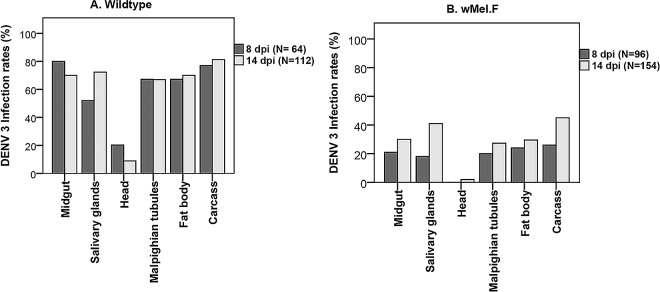
DENV infection rates in *Ae*. *aegypti* tissues for Wildtype (A) and *w*Mel.F (B) at 8 and 14 dpi.

To determine if time post infection and tissue type had an effect on DENV 3 infectivity, we examined head, salivary glands, midgut, Malpighian tubules, fat body and carcass at 8 and 14 dpi. There was a significant effect of tissue for both Wildtype (Wald = 139.60; df = 5; p< 0.0001) and *w*Mel.F (Wald = 40; df = 5; p< 0.0001) mosquitoes **(Fig 1)**. The head was the least infected tissue in both Wildtype and *w*Mel.F mosquitoes, failing to recapitulate patterns of infection in other disseminated tissues including the salivary glands. In a previous study [42] where DENV 3 infection rates in the mosquito head and body were examined, head infection rates were significantly lower than that of the body at 7 dpi in *w*Mel.F mosquitoes. However by 14 dpi in the same study there was no difference between head and body infections in *w*Mel.F mosquitoes. Furthermore, in the Wildtype mosquitoes, head infection rates were lower than that of the body at both 7 and 14 dpi but these differences were not significant [42]. The disparity observed in head infection rates between the present and previous study could possibly be due to the comparatively small sample size used by the previous study. Midgut and carcass were the most highly infected tissues in both Wildtype and *w*Mel.F mosquitoes, respectively. In Wildtype mosquitoes **(Fig 1A)** there was a significant interaction between tissue and time (Wald = 15; df = 5; p = 0.011,). Midgut infections decline with time, becoming less of a source of infection beyond 8 days. Conversely, salivary glands are still becoming increasingly infected post 8 days. Interestingly, the pattern of infection across hemocoel-associated tissues indicates early dissemination and a plateau of infection rates as well as a similarity in the capacity for these tissues to support DENV replication. There was a clear effect of time (Wald = 10; df = 5; p = 0.002) in *w*Mel.F mosquitoes with infectivity increasing from 8 to 14 dpi across all tissues **(Fig 1B).** Across the board, tissue infection rates are reduced in *w*Mel.F mosquitoes as expected [14,42] but unlike in Wildtype mosquitoes, more tissues show rising infection rates with time, suggesting the power of blocking is strongest early in infection.

### DENV load in tissues over time

**Fig 2:**
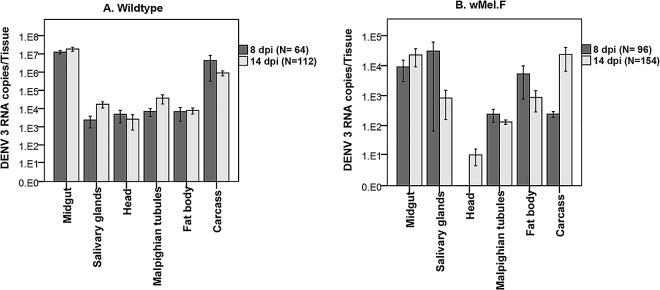
Mean ± sem DENV load in *Ae*. *aegypti* tissues for Wildtype (A) and *w*Mel.F (B) at 8 and 14 dpi.

To determine if time post infection and tissue type had an effect on DENV load in tissues, the DENV loads of all the tissues were compared at 8 and 14 dpi. There was a significant variation in DENV loads in tissues of both Wildtype (Wald = 1497; df = 5; p< 0.0001) and *w*Mel.F (Wald = 50; df = 5; p<0.0001) mosquitoes. Midguts had the highest DENV load in the Wildtype mosquitoes while head had the lowest load in the *w*Mel.F mosquitoes **(Fig 2)**. Even though time did not have a significant effect on DENV loads in tissues of both Wildtype (Wald = 2.1; df = 1; p = 0.144) and *w*Mel.F (Wald = 1.9; df = 1; p = 0.166) mosquitoes, there was a significant interaction between time and tissue for both Wildtype (Wald = 131; df = 5; p< 0.0001) and *w*Mel.F mosquitoes (Wald = 180; df = 5; p<0.0001). For instance while DENV load in the carcass decreased over time that of the salivary glands increased in Wildtype mosquitoes **(Fig 2A)**. On the other hand, DENV loads in the carcass increase over time while that of the salivary glands decreased in the *w*Mel.F mosquitoes **(Fig 2B)**. For the most part, however, DENV appears to infect tissues early, reach a peak DENV load and remain relatively stable in Wildtype mosquitoes. In general, *w*Mel.F mosquitoes, exhibited greater variation in DENV load across time and tissues and between individual mosquitoes than is seen for Wildtype possibly demonstrating variation in the efficacy of blocking.

### DENV loads in intermediate tissues predict DENV loads in Wildtype salivary glands

**Fig 3:**
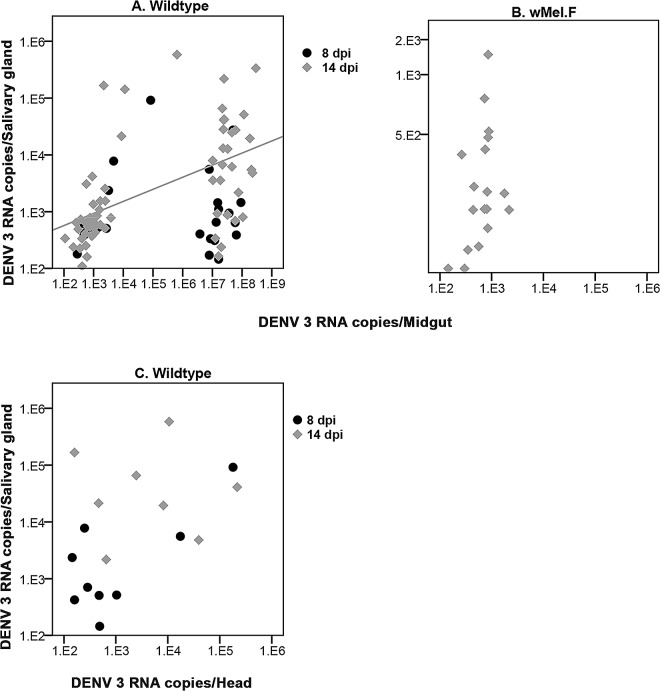
Correlation between DENV 3 load in salivary gland and that of midgut and head in *Ae*. *aegypti*. **(A)** Correlation significant at 14 dpi only (R^2^ Linear = 0.28 at 14 dpi) for Wildtype midgut. **(B)** No correlation at 14 dpi for *w*Mel.F midgut. **(C)** No correlation at either 8 or 14 dpi for Wildtype head.

**Fig 4:**
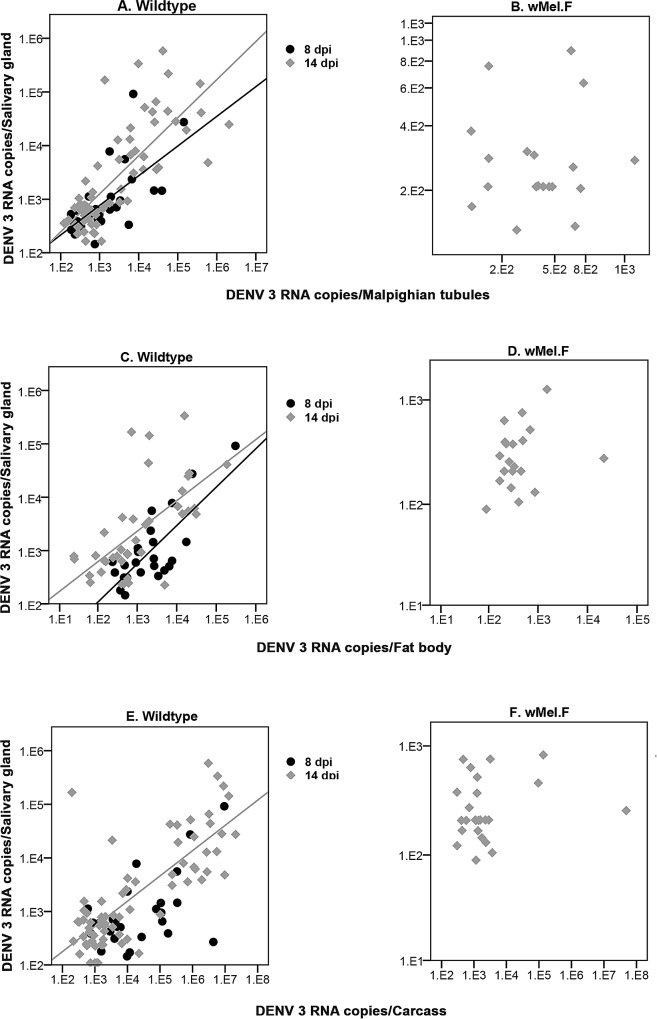
Correlation between DENV load in salivary gland and Malpighian tubules, fat body and carcass and in *Ae*. *aegypti*. **(A)** Correlation significant at both 8dpi (R^2^ Linear = 0.428) and 14dpi (R^2^ Linear = 0.582) for Wildtype Malpighian tubules. **(B)** No correlation at 14 dpi for *w*Mel.F Malpighian tubules. **(C)** Significant correlation at both 8dpi (R^2^ Linear = 0.597) and 14dpi (R^2^ Linear = 0.399) for Wildtype fat body. **(D)** No correlation at 14 dpi for *w*Mel.F fat body. **(E)** Significant correlation at14 dpi only (R^2^ Linear = 0.593) for Wildtype carcass. **(F)** No correlation at 14 dpi for *w*Mel.F carcass.

We examined if DENV load in a range of tissues early in the infection process was predictive of loads in the salivary glands by testing for correlations. In the *w*Mel.F mosquito line, the efficacy of blocking effect rendered many mosquitoes uninfected. As such sufficient numbers of DENV positive heads were not obtained for either time point and all tissues at 8 dpi had to be excluded. For Wildtype mosquitoes head DENV loads were not predictive of salivary gland DENV loads at either time point, 8dpi (r = 0.250; p = 0.516) or 14dpi (r = -0.071; p = 0.867) **(Fig 3C).** There was a significant correlation between midgut and salivary gland DENV loads only at 14 dpi (r = 0.701; p<0.0001) **(Fig 3A)** in the Wildtype but not in the *w*Mel.F mosquitoes (r = 0.460; p = 0.550) at 14 dpi **(Fig 3B).** In the Wildtype, salivary glands DENV loads were positively correlated to that of the Malpighian tubules at both 8 (r = 0.684; p<0.0001) and 14 (r = 0.783; p<0.0001) dpi **(Fig 4A)**. A positive correlation was also found between fat body and salivary gland DENV loads at both 8 (r = 0.594; p = 0.002) and 14 (r = 0.684; p<0.0001) dpi **(Fig 4C)**. Carcass DENV loads were positively correlated to salivary gland DENV loads only at 14 dpi (r = 0.701; p<0.0001) **(Fig 4E)**. Malpighian tubule (r = -0.260; p = 0.917), fat body (r = 0.299; p = 0.188) and carcass (r = 0.127; p = 0.545) DENV loads were not predictive of DENV loads in *w*Mel.F mosquito salivary gland **(Fig 4B, Fig 4D and Fig 4F)**. In summary, these findings show that DENV load in upstream tissues may predict salivary gland loads in Wildtype but not *w*Mel.F infected *Ae*. *aegypti*.

### *Wolbachia* density in tissues over time

**Fig 5:**
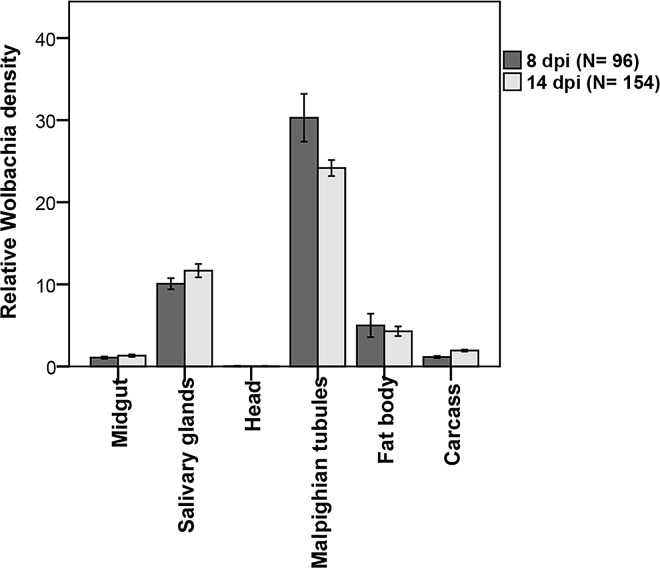
*Wolbachia* tissue densities in *Ae aegypti*. Malpighian tubules had the highest density and head the lowest density.

To determine if time and tissue type affect *Wolbachia* density in *w*Mel.F mosquitoes, we compared *Wolbachia* density in the head, salivary glands, midgut, Malpighian tubules, fat body and carcass over two time points (8 and 14 dpi) **(Fig 5).** We observed that time had no effect (Wald = 0.18; df = 1; p = 0.671) on *Wolbachia* density in tissues. There was a significant tissue effect (Wald = 3423; df = 5; p<0.0001) demonstrating that *Wolbachia* density varied across tissue types. For instance, *Wolbachia* was most abundant in the Malpighian tubules with the head having the lowest bacterial density. There was an interaction between time and tissue type (WALD = 28; df = 5; p<0.0001). For example in the Malpighian tubules, *Wolbachia* density decreased from 8 dpi to 14 dpi while that of the carcass increased from 8 to 14 dpi **(Fig 5).** In summary, *Wolbachia* density varied across different tissue types with the Malpighian tubules and head harbouring the highest and the least number of *Wolbachia* respectively.

### Correlation between *Wolbachia* density and DENV load in individual tissues

**Fig 6:**
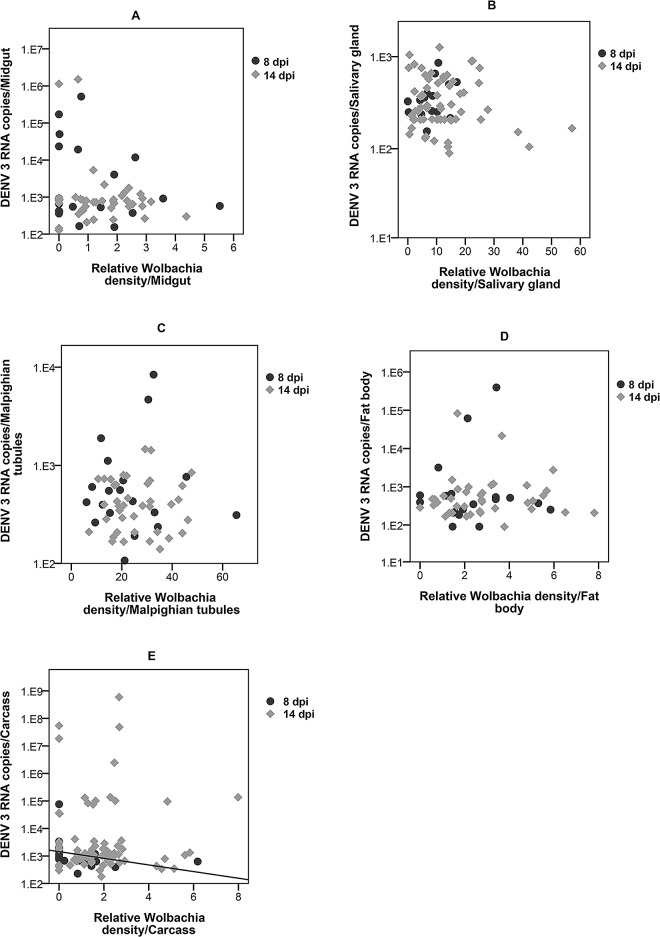
Correlation between *Wolbachia* density and DENV load in *w*Mel.F tissues. No correlation between *Wolbachia* density and DENV load in the midgut **(A)**, salivary glands **(B)** Malpighian tubules **(C)**, fat body **(D)** at 8 and 14 dpi. **(E)** DENV load decreased as *Wolbachia* density increased (Linear R^2^ = 0.125) in the carcass at 8 dpi but not at 14dpi.

We first examined if the *Wolbachia* density in particular tissues was predictive of DENV load in that same tissue. In the carcass *Wolbachia* density was negatively correlated (r = -0.580; p = 0.005) with DENV load at 8 dpi, but this was not the case at 14 dpi (r = 0.160; p = 0.898) **(Fig 6E).** At 8 dpi *Wolbachia* density in the midgut (r = -0.125; p = 0.601), salivary glands (r = 0.453; p = 0.0680), Malpighian tubules (r = -0.095; p = 0.700) and fat body (r = 0.070; p = 0.765) were not correlated with DENV load **(Fig 6A–6D)**. Neither was there a significant correlation between *Wolbachia* density in the midgut (r = -0.060; p = 0.702), salivary glands (r = -0.063; p = 0.626), Malpighian tubules (r = -0.026; p = 0.873) and fat body (r = 0.0044; p = 0.784) and DENV load at 14 dpi **(Fig 6A–6D).** Infection rates for both DENV and *Wolbachia* were too low to be statistically analysed in the head for both 8 and 14 dpi. These findings demonstrate that *Wolbachia* density is not predictive of DENV load within any of tissue types tested.

### Correlation between *Wolbachia* density in intermediate tissues and DENV load in salivary glands

**Fig 7:**
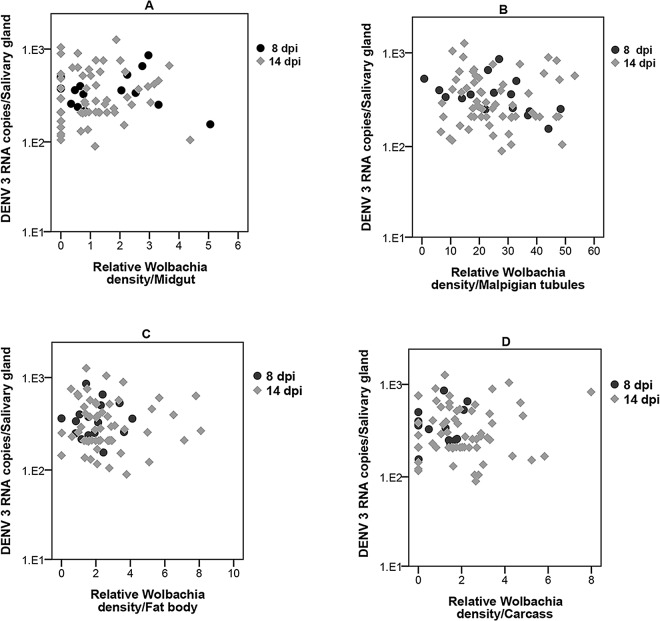
Correlation between *Wolbachia* density in tissues and DENV loads in salivary glands. No correlation between salivary glands DENV loads and *Wolbachia* density in **(A)** midgut, **(B)** Malpighian tubules, **(C)** fat body, and **(D)** carcass.

To determine if *Wolbachia* density in any particular tissue has an effect on DENV load in the salivary gland, we compared *Wolbachia* density in all the five dissected tissues to DENV load in the salivary glands. At 8 dpi there was no correlation between *Wolbachia* density in midgut (r = 0.21; p = 0.940), Malpighian tubules (r = 0.412; p = 0.101), fat body (r = 0.221; p = 0.395) and carcass (r = 0.051; p = 0.844) and DENV load in the salivary glands **(Fig 7A–7D)**. Neither was there a significant correlation at 14 dp between the *Wolbachia* density in midgut (r = 0.155; p = 0.232), Malpighian tubules (r = 0.046; p = 0.732), fat body (r = 0.060; p = 0.642) and carcass (r = 0.32; p = 0.801) **(Fig 7A–7D)**. Infection rates for both *Wolbachia* and DENV were too low to be statistically analysed in the head at both 8 and 14 dpi. These results show that *Wolbachia* density in intermediate tissues does not predict salivary gland DENV load.

## Discussion

This study investigated whether particular tissues, especially those with high *Wolbachia* densities or immune function, are important in *Wolbachia*-mediated DENV blocking. Specifically, we assessed whether DENV loads or *Wolbachia* densities encountered in intermediate tissues predict subsequent infection in the salivary glands. All tissues examined were susceptible to DENV 3 infection. As expected, the tissues of *Wolbachia* infected mosquitoes had considerably lower DENV infection rates compared to the Wildtype, due to pathogen inhibition [11,14,42]. Interestingly, we observed that the strength of blocking was strongest early in the infection process but then declined with time. This is consistent with observations in *Wolbachia* infected flies [29] where DCV numbers are initially low but progressively climb from 2 to 30 days post infection. Moreira *et al*., and Ye *et al*., [14,44] also observed slight increases in infection rates over time in *w*MelPop-CLA and *w*Mel infected mosquitoes respectively, but only when DENV titres in the blood meal were high. The differences in blocking between early and late stages of infection raise the question whether the midgut may be playing a particular role in *Wolbachia*-DENV inhibition.

The midgut, not surprisingly had a high initial infection rate in Wildtype mosquitoes but as observed in other studies [41], the infection rate declined as the salivary glands became increasingly infected. This increasing infection suggests that salivary glands may be a site of DENV replication as well as accumulation. The very low infection rates in the head for both Wildtype and *w*Mel.F mosquitoes were unexpected due to the fact that the head is generally used as a proxy for DENV dissemination. Our finding is contrary to previous studies that found higher head infection using immunofluorescence assays and RNA estimates of DENV 1, 2 and 3 [39,41,50]. However infection rates may be affected by the specific DENV and mosquito genotypes studied [51] or environmental effects that often vary across studies [52,53]. Lastly, head infection rates could also be inflated if head tissues become contaminated with the salivary gland during dissections.

While it is known that infection of the midgut is dependent on the amount of DENV the mosquito ingests [54], whether this affects virus dissemination from the midgut to other tissues including the salivary glands is not well understood. Salazar *et al*., [41] have reported that the trachea may facilitate DENV 2 dissemination from the midgut. However other studies suggest that virus disseminates to mosquito tissues through the hemolymph [55,56]. DENV loads in almost all hemocoel-associated tissues of Wildtype mosquitoes were similar to one another and predictive of salivary gland loads at 8 dpi. This pattern suggests that DENV infects these tissues in parallel and then seeps into the hemolymph and then makes its way to the salivary glands. Interestingly, midgut DENV loads in Wildtype mosquitoes were only predictive of salivary gland DENV loads late in the infection process. This is likely explained by incomplete dissemination of DENV out of the midgut in the early stages of infection.

Our findings demonstrate that the infection dynamics of the fat body is not different from other hemocoel-associated tissues in Wildtype mosquitoes. This finding does not support a special role of this immune active tissue [34,57] in DENV inhibition. Innate immune genes, particularly in the TOLL pathway, have been shown to decline in the fat body after 3 days when challenged with DENV [33]. Therefore the capacity for DENV to successfully infect this tissue may reflect declines in transcription of immunity genes over time. Another tissue that has been reported to be involved in insect immunity is the Malpighian tubules. In *Drosophila* Malpighian tubules exhibit basal expression of antimicrobial peptides (AMPs) that then increase in response to immune challenge [58]. Malpighian tubules have further been shown to fight infection independent of the fat body in *Drosophila* [59] and melanise larvae of the dog heartworm, *Dirofilaria immitis* in *Ae*.*sollicitans* [60]. Regardless, the Malpighian tubules had similar DENV loads to other tissues in Wildtype mosquitoes. This suggests that the Malpighian tubules are unlikely to be playing an immune role in modulating DENV replication and transmission in *Ae*. *aegypti*.

Unlike in Wildtype mosquitoes, DENV loads in the *w*Mel.F mosquito midgut, Malpighian tubules, fat body and carcass did not significantly influence that of the salivary glands. There was no correlation between DENV load in the salivary glands and *Wolbachia* density in any of the tissues studied. In almost all cases, *Wolbachia* densities in particular tissues were also not predictive of DENV loads in those same tissues. This is true even for the Malpighian tubules that harbour extremely high densities of *Wolbachia*. This unique tropism may relate to access to nitrogen given *Wolbachia’s* reliance on host amino acids for nutrition [61]. Our findings are contrary to those for *Drosophila* [32] where *Wolbachia* density in the head, gut, and Malpighian tubules correlated with DCV inhibition in the whole fly. *Drosophila*, however, is naturally infected with *Wolbachia* unlike *Ae*. *aegypti* [11]. Native hosts for *Wolbachia* appear to have more restricted tissue distributions and reduced bacterial densities compared to novel hosts that may be the result of coadaptation [62]. Therefore the relationships between tissue densities and blocking in flies may not be the same as in novelly infected mosquitoes.

Across a range of studies *Wolbachia* density appears to correlate with the strength of DENV blocking/viral inhibition [11,26–29]. There are several possible models for this relationship. Firstly, in the simplest case there is a negative linear relationship between the two. In whole mosquitoes this hypothesis is supported based on a comparison of DENV blocking between the *w*MelPop strain, which grows to very high densities, and the *w*Mel strain, which grows to moderate densities [11]. Secondly, blocking may become apparent only after particular thresholds of *Wolbachia* densities are reached. In an *Ae*. *albopictus* cell line, a minimum density of ~960 *Wolbachia* per host cell (wsp/actin) was required for complete blocking of DENV [28] with no obvious correlations at lower *Wolbachia* densities. We observed the highest density of ~30 *Wolbachia* per host cell (WD0513/RPS17) in the Malpighian tubules demonstrating that *Wolbachia* does not normally grow to such high densities in *w*Mel.F *Ae aegypti* tissues. Our work therefore confirms the observations in cell lines [28] that at lower densities, there is no correlation between *Wolbachia* densities and blocking. Given different functional roles of particular tissues, especially with regards to immunity, we also hypothesized there may be tissue specific contributions to DENV blocking. Our work suggests, however, that none of the tissues we examined played a greater role in the expression of blocking. Instead, efficacy of blocking may be determined at the level of the individual cell. Our work does not rule out the involvement of immunity[19–21] or nutrient competition for key resources [22] in the mechanism of inhibition, but suggests *Wolbachia* must act through aspects of host cell biology that are either systemic or fundamental to diverse cell types.

### Conclusion

Our findings in Wildtype mosquitoes demonstrate that DENV disseminates from the midgut and infects mosquito hemocoel-associated tissues equally through time. They also suggest that infection of the mosquito head is not an accurate proxy for the assessment of dissemination. In terms of *Wolbachia*-based blocking of DENV this study reports two main findings. Firstly, the *Wolbachia* tissue densities in the mosquito are not linear predictors of DENV load as has been reported in cell lines where densities are usually very high. This may be related to the much lower densities naturally present in insect tissues. Secondly, DENV inhibition is unlikely to be explained by tissue specific mechanisms. Future studies seeking to dissect the involvement of either immunity, resource competition or other unknown contributors to mechanism, should focus on aspects of host cell biology that are fundamental across tissues. Generalisations from cell line based-studies are likely to be more biologically meaningful when *Wolbachia* densities are lower and more reflective of those found in insect tissues.
